# Supplementary figures and images for: Induction of fatigue-like behavior by pelvic irradiation of male mice alters cognitive behaviors and BDNF expression
Source: PLoS One. 2020 Jul 2;15(7):e0235566. doi: 10.1371/journal.pone.0235566 (PMC7332074; doi:10.1371/journal.pone.0235566)

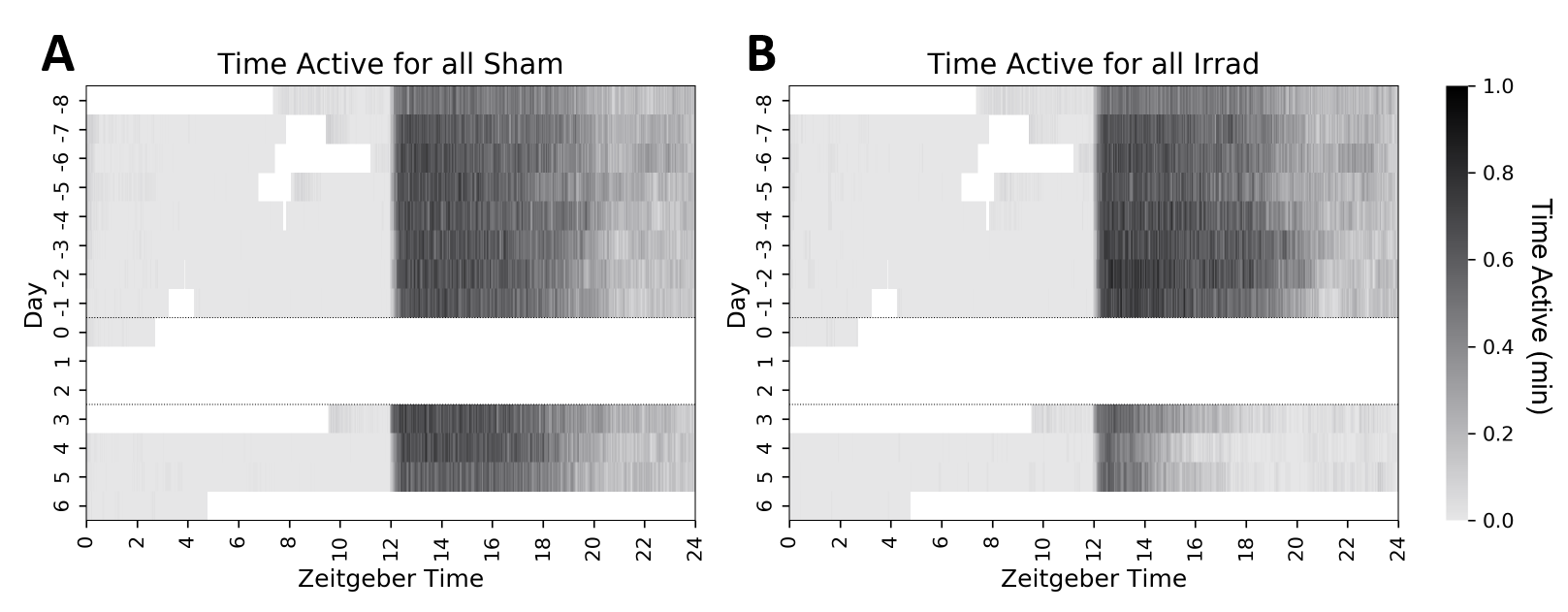

Supplement: S1 Fig — Mean VWRA time active for each minute of recording, with darker colors representing more activity, light colors representing lower levels of activity, and white representing no data. Irradiation took place on days 0, 1, and 2. Zeitgeber time is the number of hours after lights are turned on at 6 a.m. (TIF) [file pone.0235566.s001.tif]

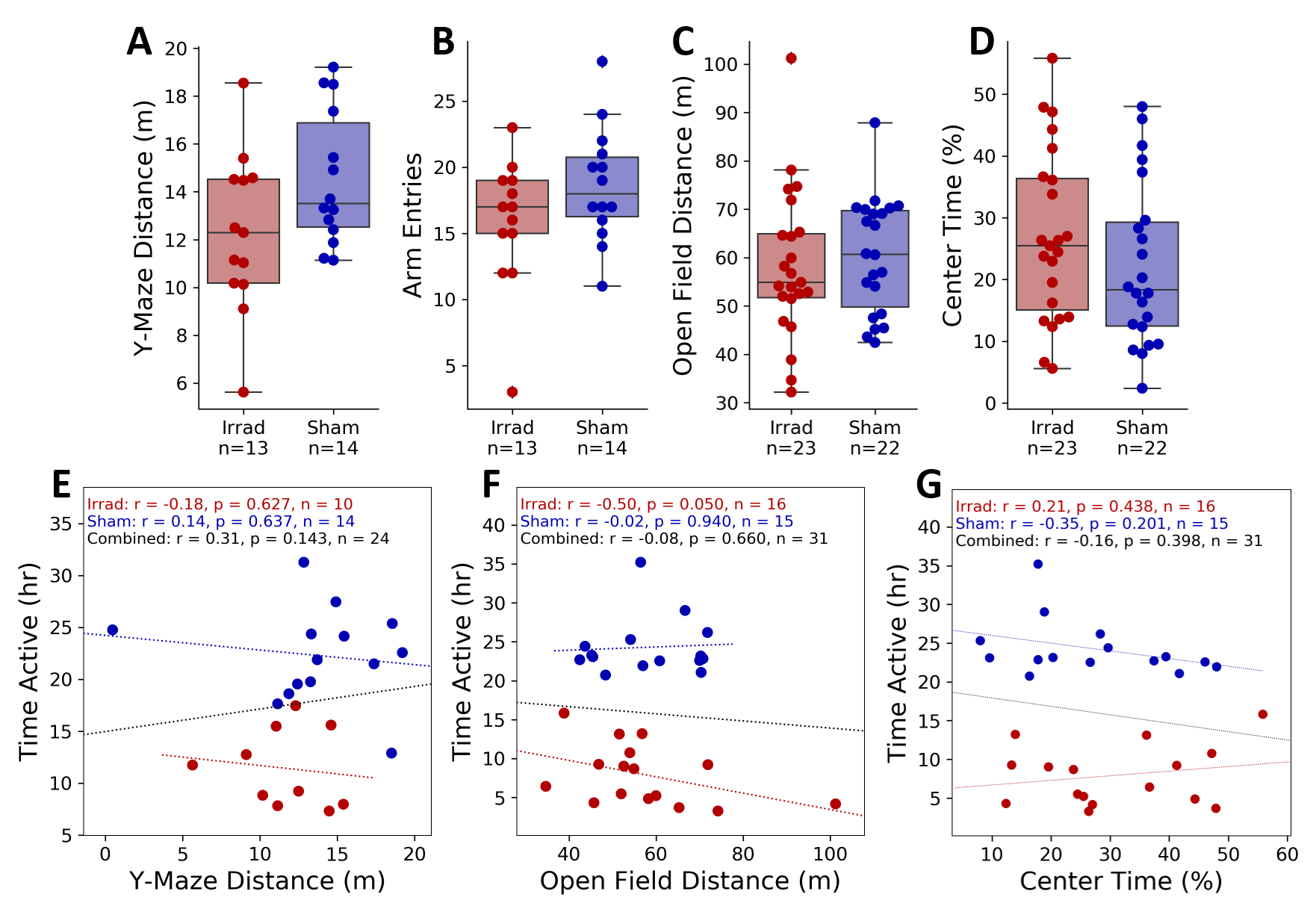

Supplement: S2 Fig — (A) Distance travelled in the 5-minute Y-maze test. (B) The total number of arm entries during the Y-maze were not significantly affected by irradiation (d = 0.60, t = 1.56, p = 0.132, n = 27). (C) Distance travelled in the 30-minute open field test. (D) Center time in the open field test. (E) There were no significant correlations between VWRA and distances travelled in the Y-maze. (F–G) There were no significant correlations between VWRA and distances travelled (F) or open time (G) in the open field. (TIF) [file pone.0235566.s002.tif]

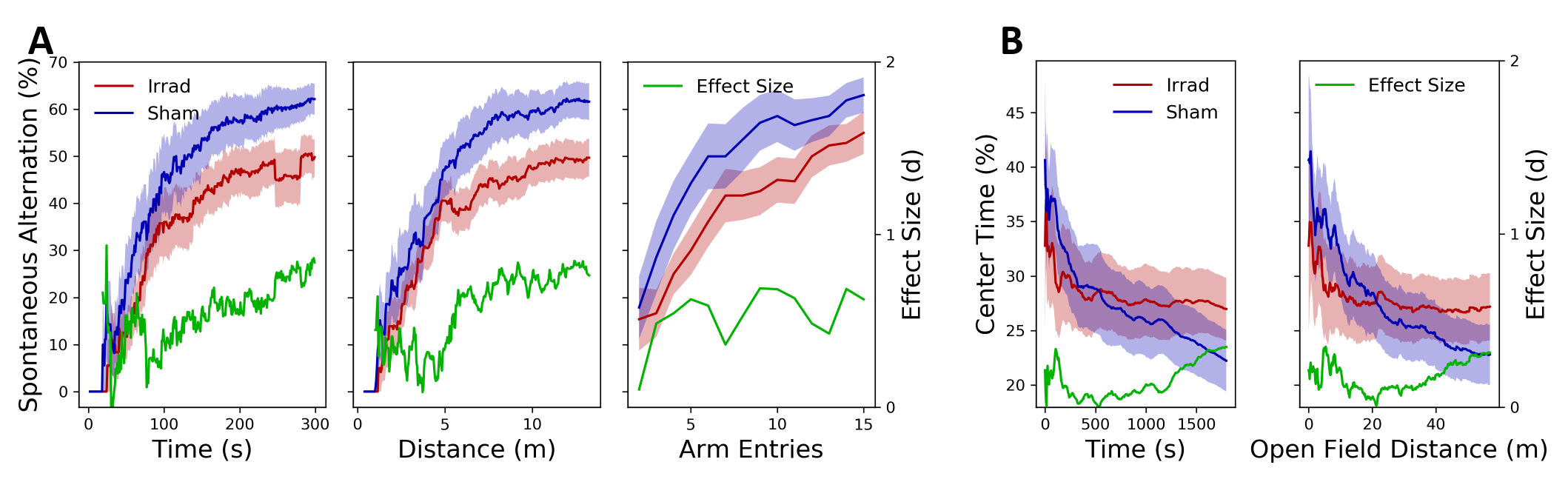

Supplement: S3 Fig — Since irradiation showed different effects on reversal learning over time vs. over participation with the task, we did a similar analysis on the arena behaviors. (A) Plotting spontaneous alternation behavior over time in the Y-maze, distance travelled, or total arm entries had little effect on the appearance of the plots. (B) Plotting center time in the open field over time in the arena or over distance traveled had little effect on the appearance of the plots. The effect size is plotted with a green line without shading and uses the right-hand axis labels. (TIF) [file pone.0235566.s003.tif]

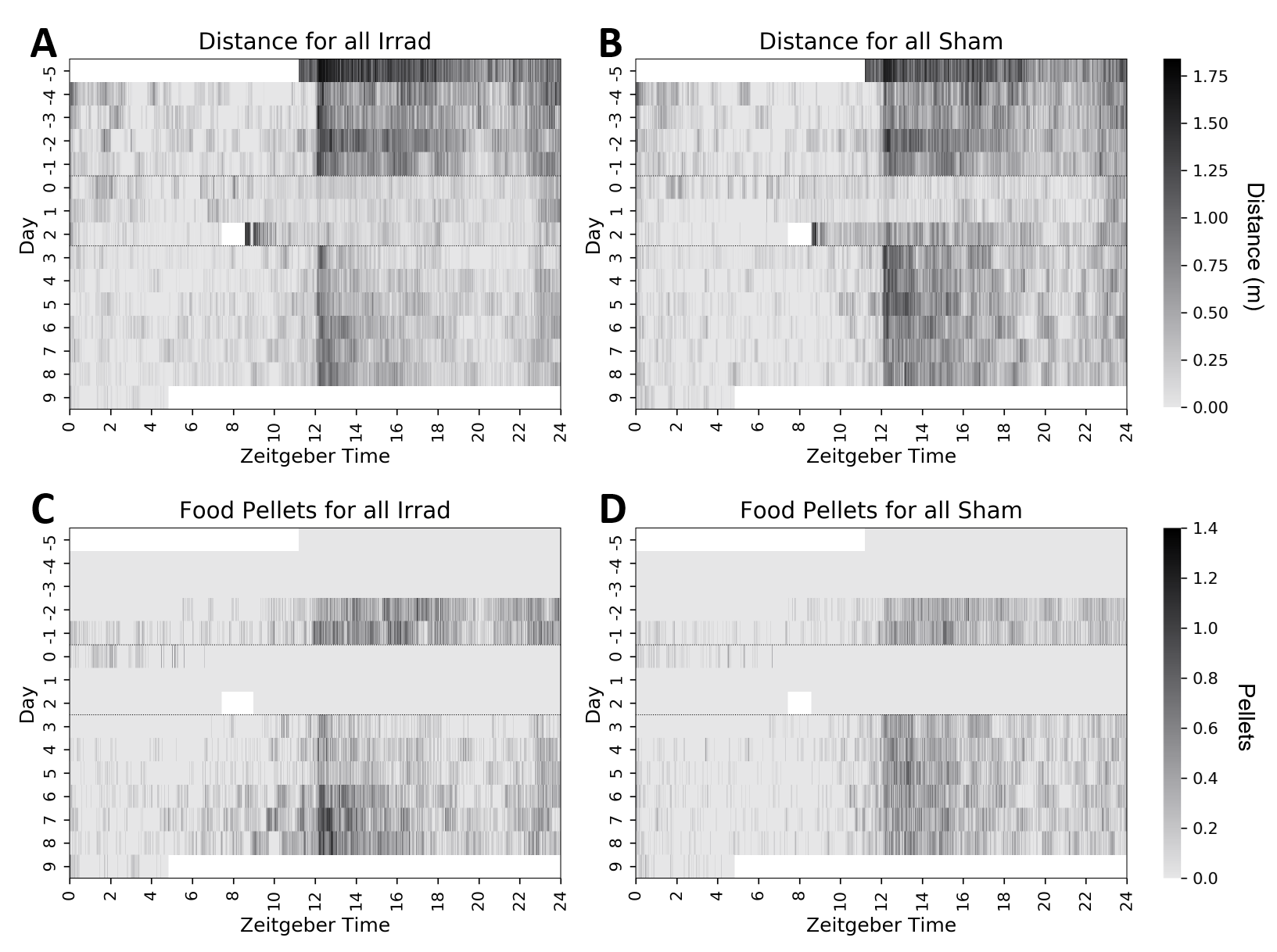

Supplement: S4 Fig — (A–B) Mean locomotor distance totals for each minute of recording, with darker colors representing greater distances, light colors representing lower lesser distances, and white representing no data. (C–D) Mean number of food pellets dispensed during each minute of recording, with darker colors representing more pellets, light colors representing fewer pellets, and white representing no data. Irradiation took place on days 0, 1, and 2, and no food pellets were dispensed during this time (mice had ad libitum access to chow). Zeitgeber time is the number of hours after lights are turned on at 6 a.m. (TIF) [file pone.0235566.s004.tif]

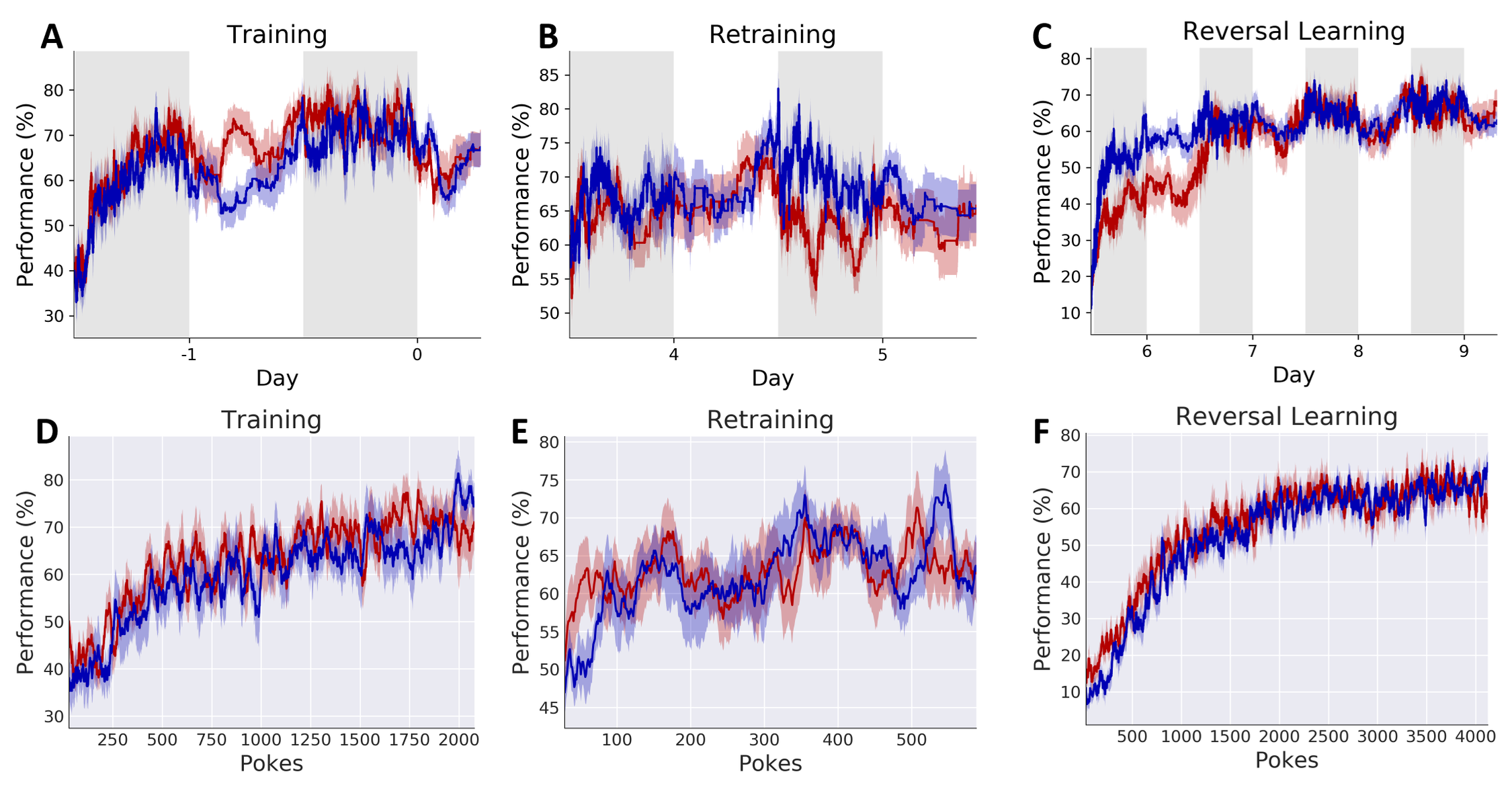

Supplement: S5 Fig — (A–C) Performance over time during training (A), retraining (B), and reversal learning (C). Dark shading represents the dark cycle (night). (D-F) Performance over the total number of pokes during training (D), retraining (E), and reversal learning (F). (TIF) [file pone.0235566.s005.tif]

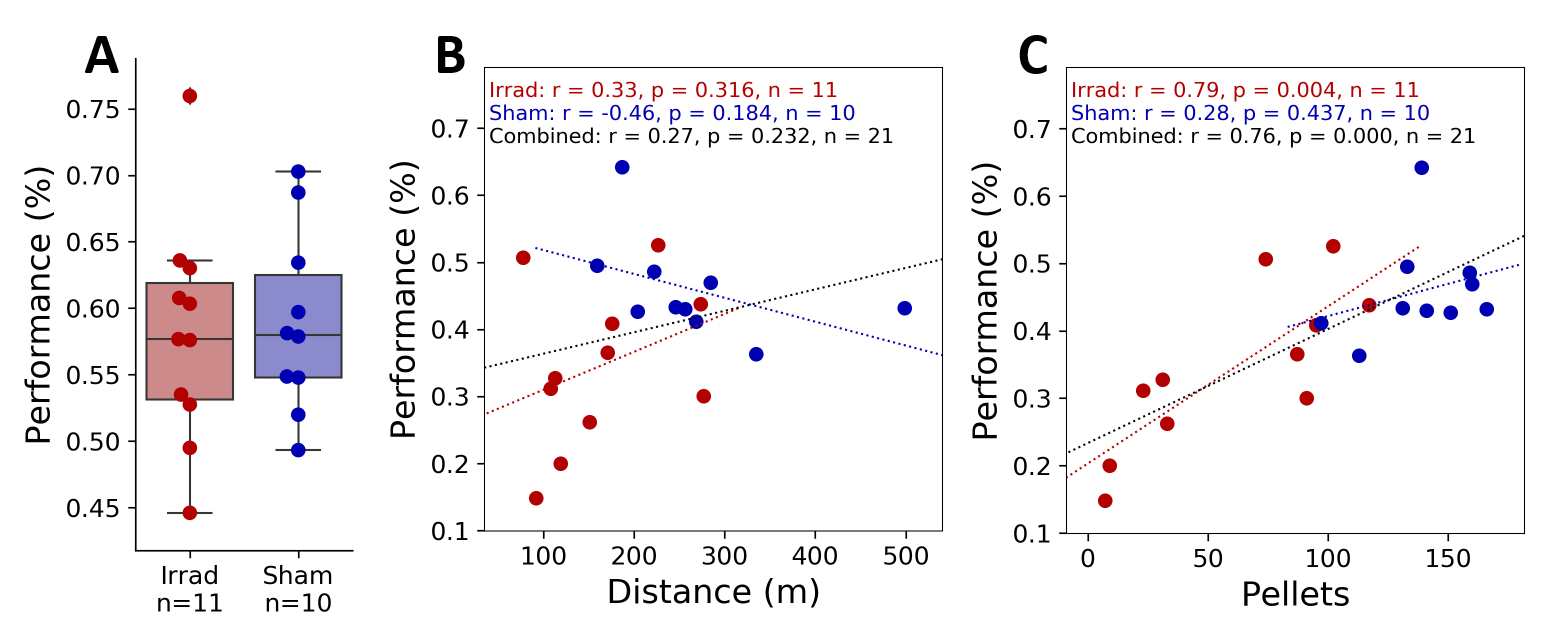

Supplement: S6 Fig — (A) Mean performance cumulative over the first 1536 pokes of reversal learning, which is the mean number of nose-pokes over the first night across all mice in the Sham group. (B) There were no significant correlations between distance traveled and performance during the first night of reversal learning. (C) There were large significant correlations between food pellets dispensed and performance during the first night of reversal learning, particularly for the Irrad group. This is not surprising, as performance at the task causes food pellets to dispense. (TIF) [file pone.0235566.s006.tif]

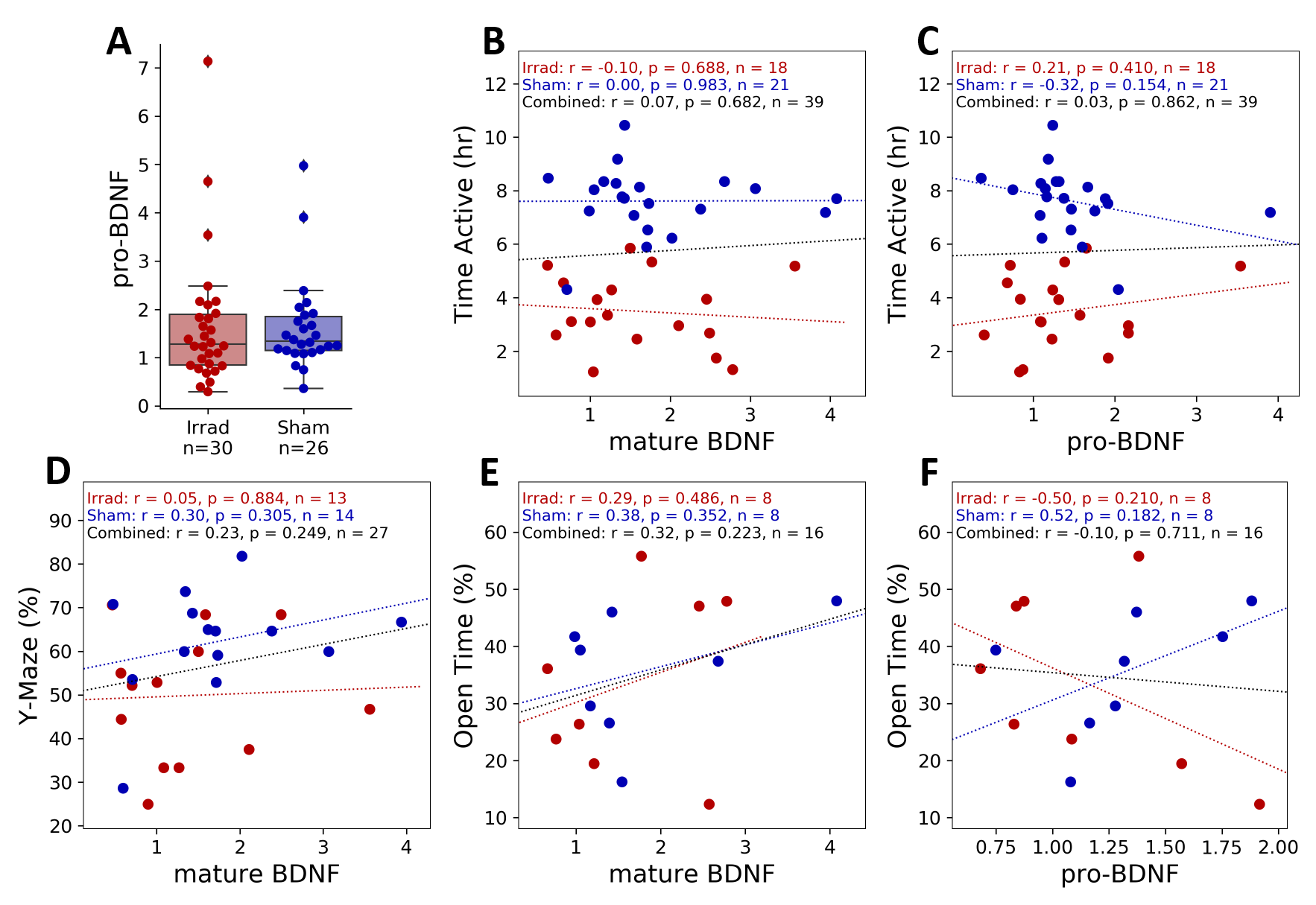

Supplement: S7 Fig — (A) Densitometric analysis results of proBDNF normalized to GAPDH. (B–C) There were no significant correlations between mBDNF or proBDNF and VWRA. (D) There were no significant correlations between mBDNF and spontaneous alternation in the Y-maze. (E–F) There were no significant correlations between mBDNF or proBDNF and open time in the open field test. (TIF) [file pone.0235566.s007.tif]
