# Supplementary material for: Induction of fatigue-like behavior by pelvic irradiation of male mice alters cognitive behaviors and BDNF expression
Source: PLoS One. 2020 Jul 2;15(7):e0235566. doi: 10.1371/journal.pone.0235566 (PMC7332074; doi:10.1371/journal.pone.0235566)

I=Irradiation
S=Sham
B=Blank
P=Pooled

Gel 1 probed for BDNF

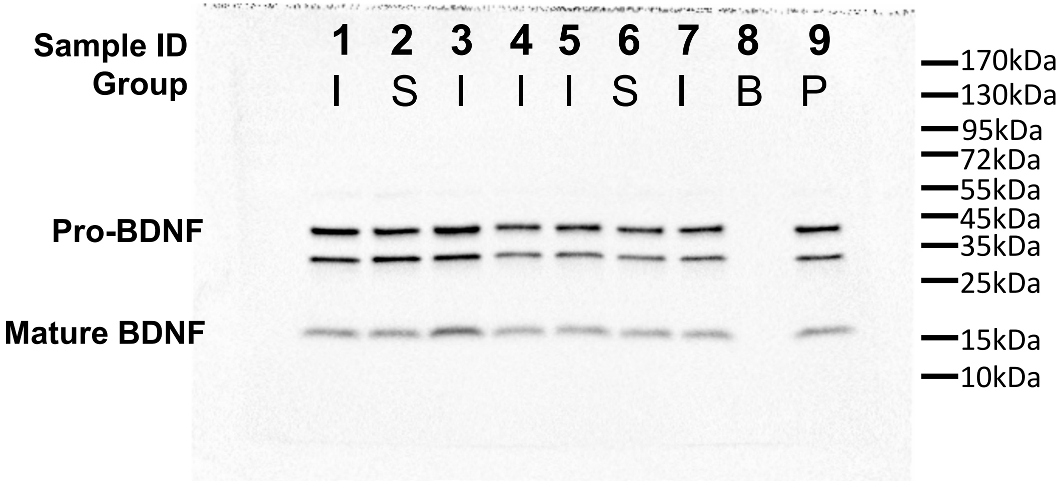

Gel 1 reprobed for GAPDH

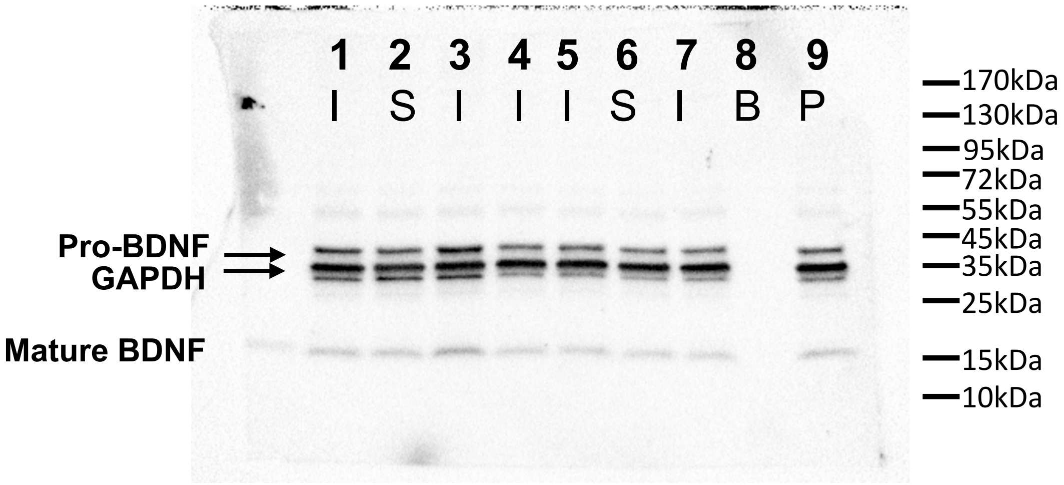

Gel 2 probed for BDNF

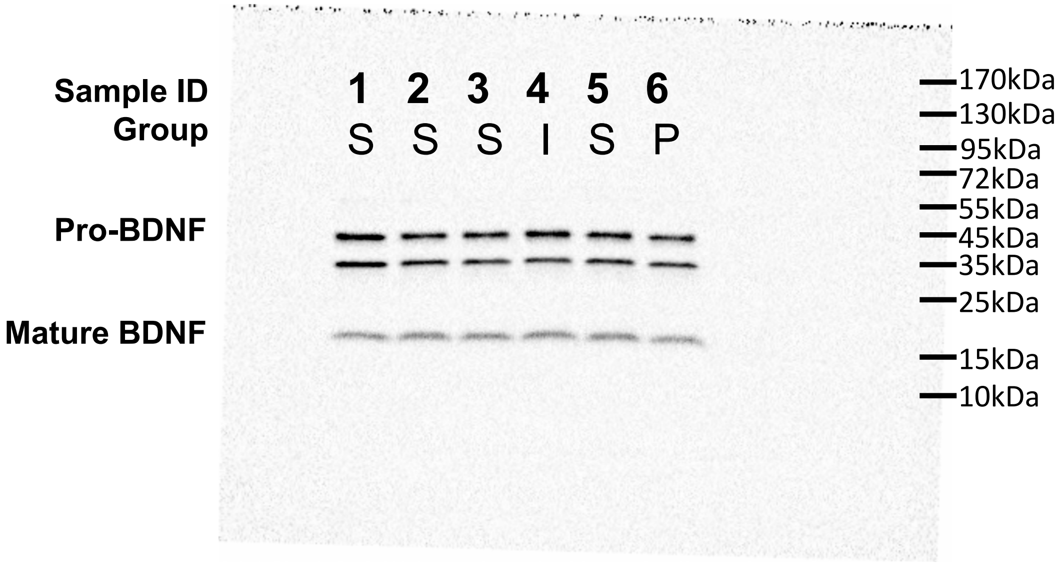

Gel 2 reprobed for GAPDH

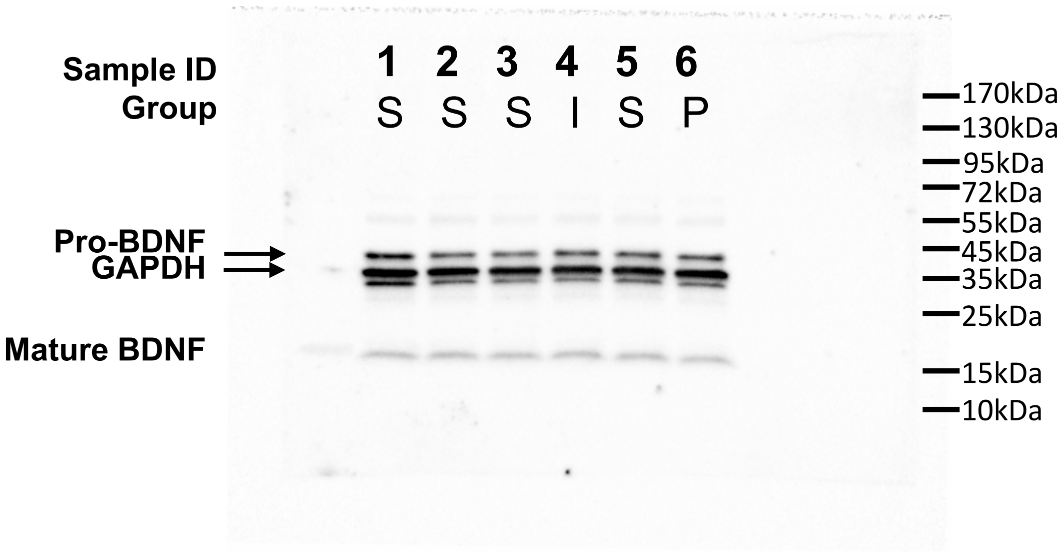

Gel 3 probed for BDNF

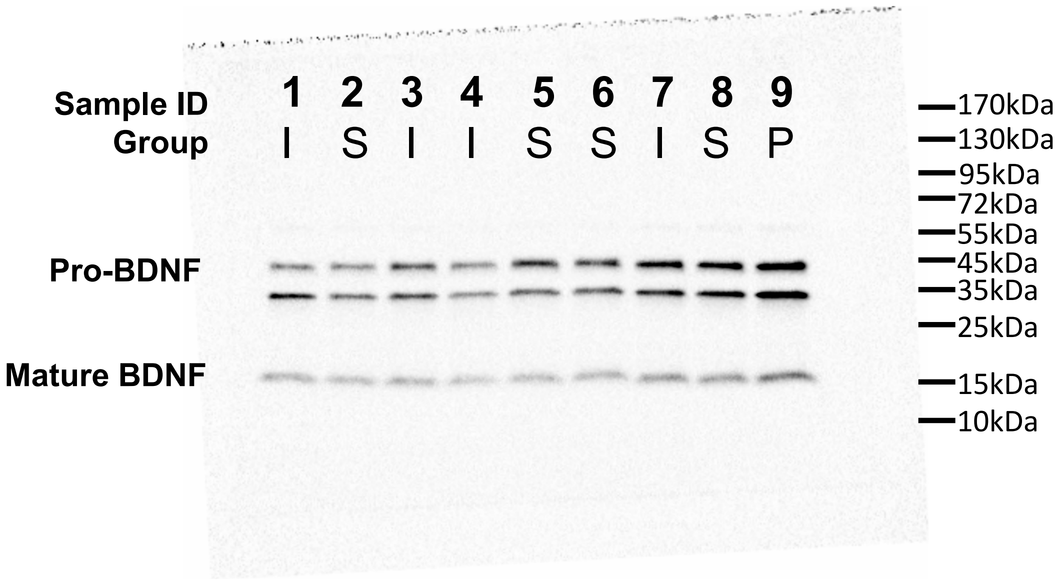

Gel 3 reprobed for GAPDH

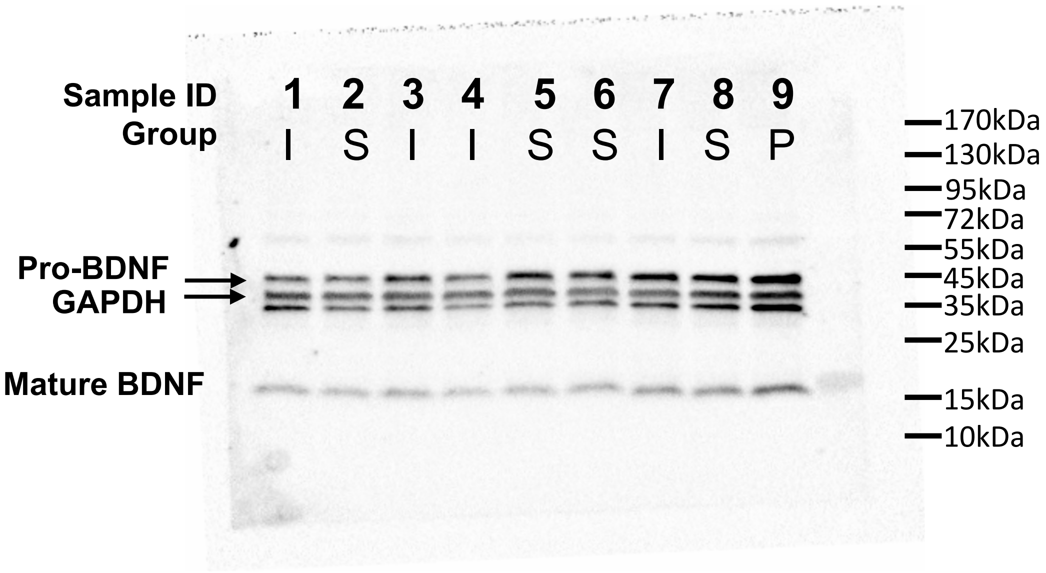

Gel 4 probed for BDNF

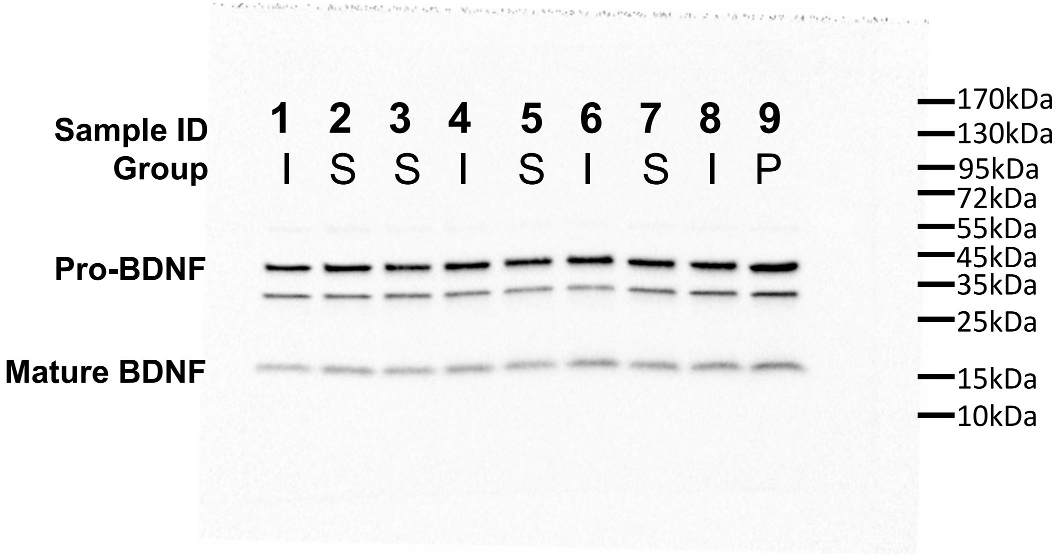

Gel 4 reprobed for GAPDH

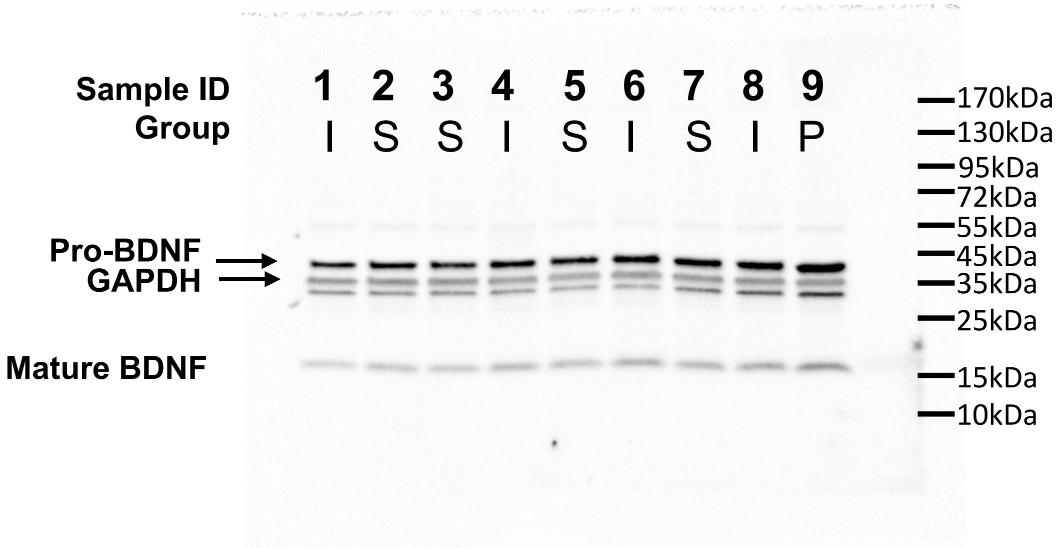

Gel 5 probed for BDNF

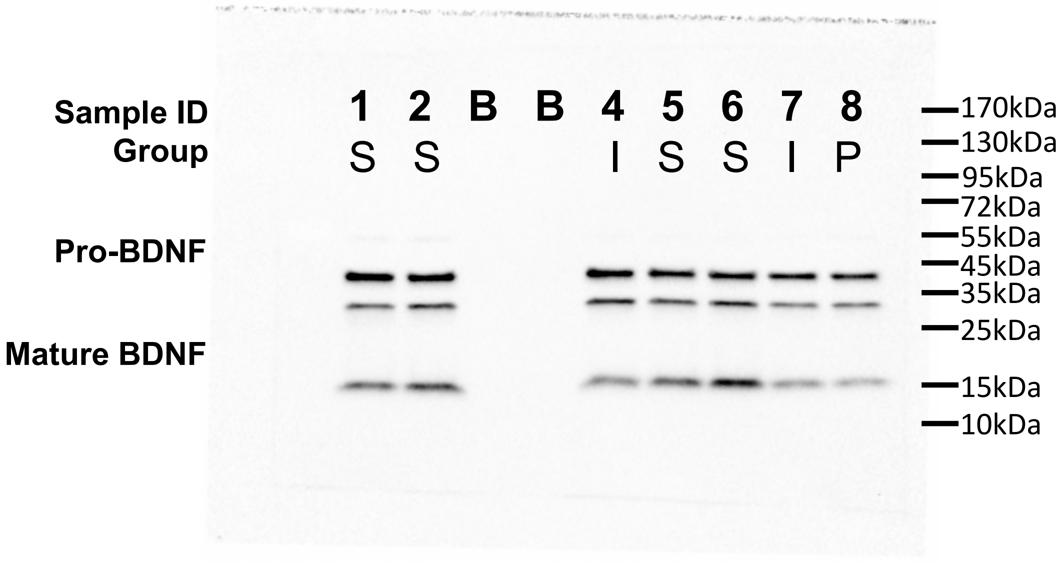

Gel 5 reprobed for GAPDH

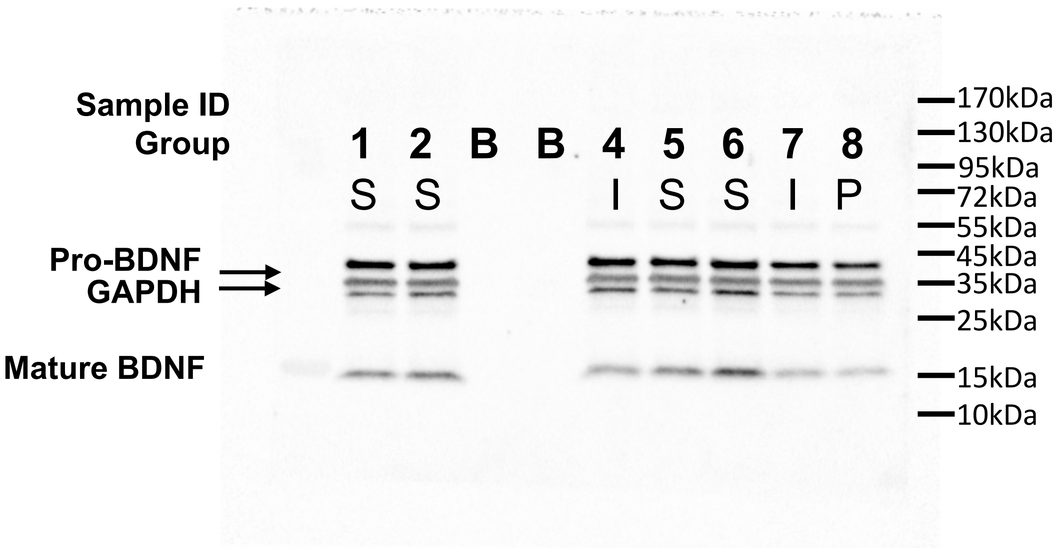

Gel 6 probed for BDNF

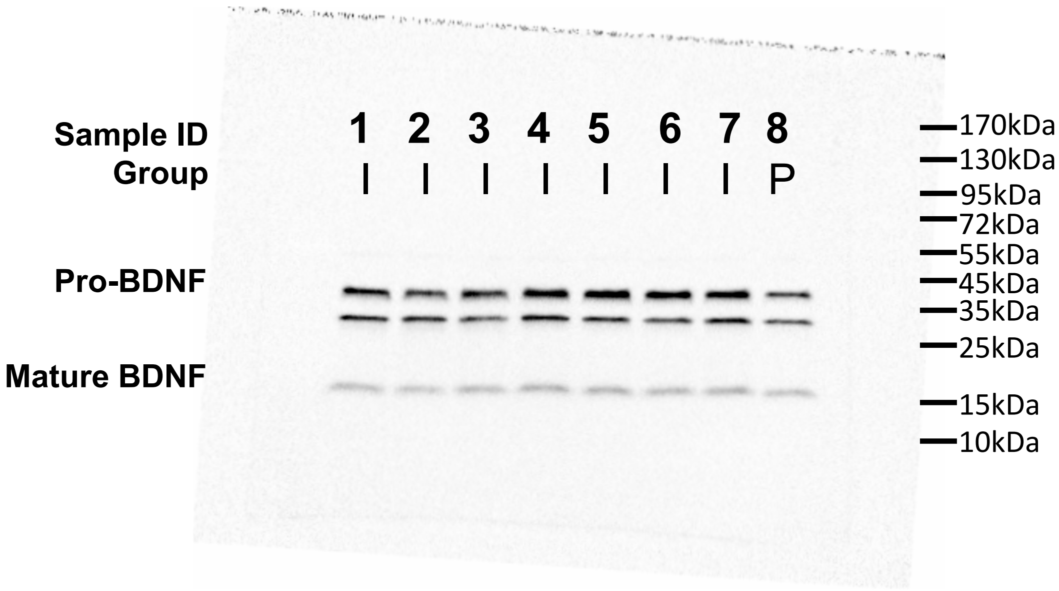

Gel 6 reprobed for GAPDH

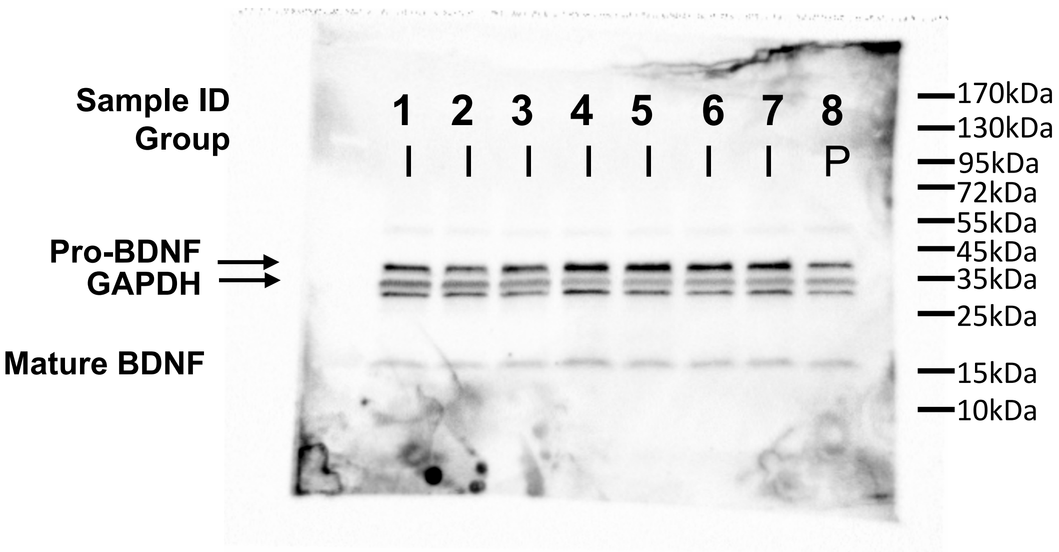

Gel 7 probed for BDNF

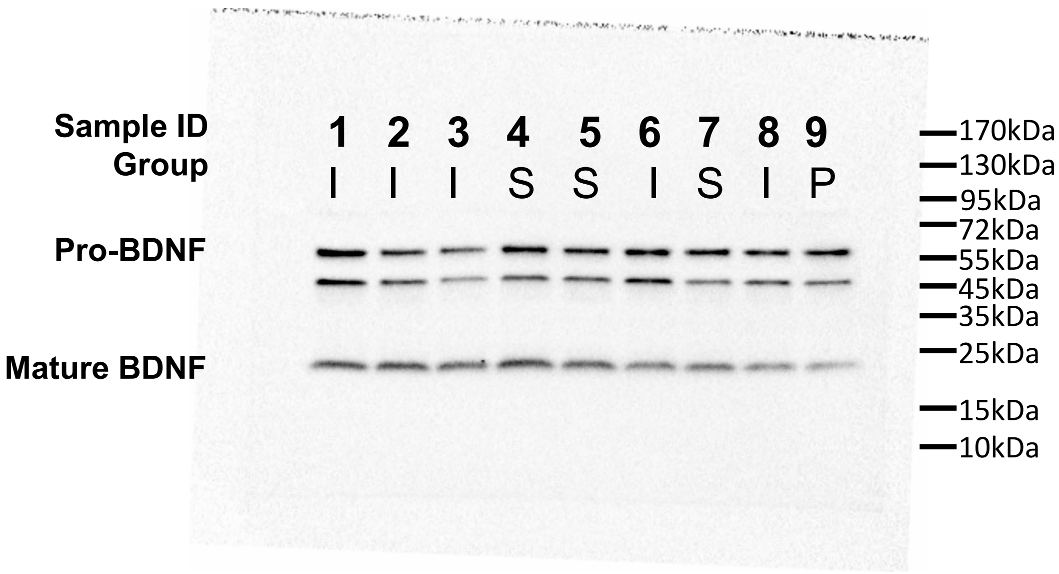

Gel 7 reprobed for GAPDH

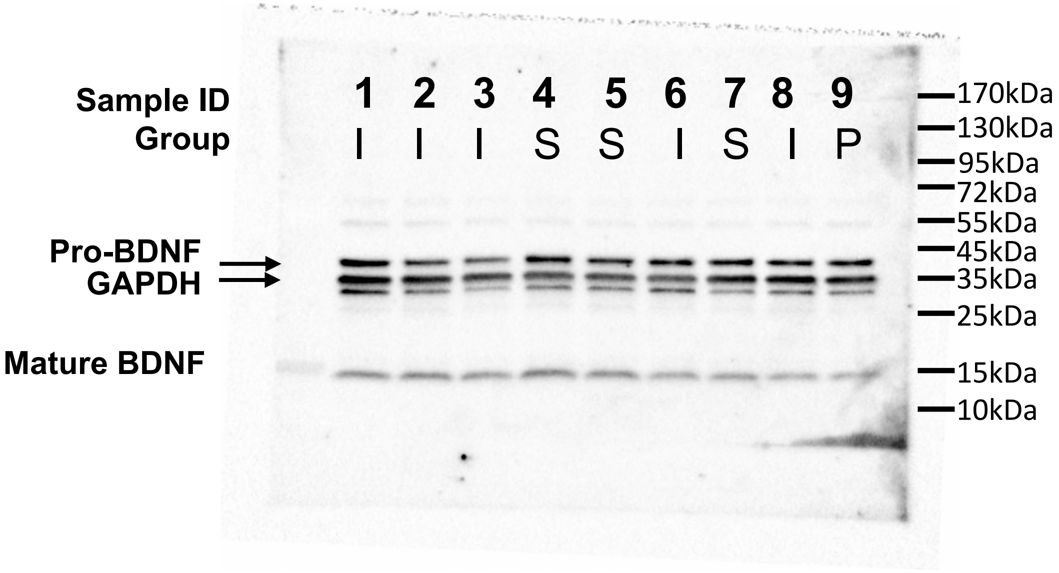

Gel 8 probed for BDNF

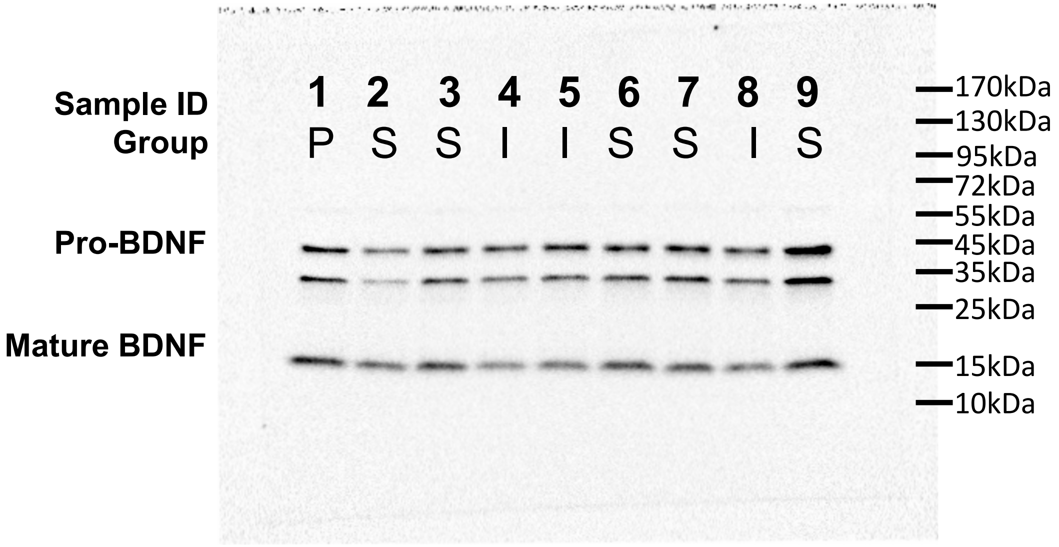

Gel 8 reprobed for GAPDH

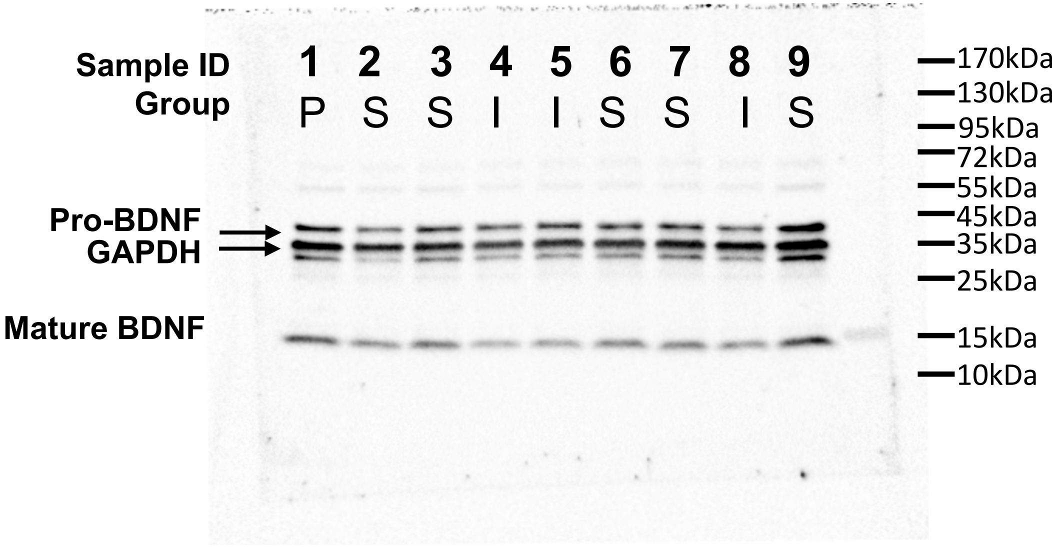

Gel 9 probed for BDNF

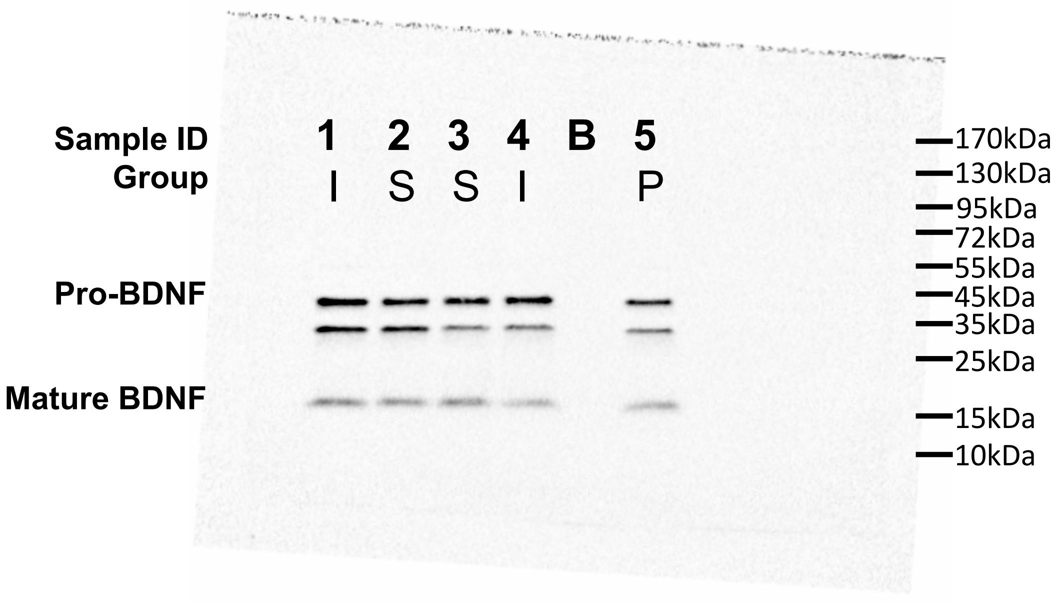

Gel 9 reprobed for GAPDH

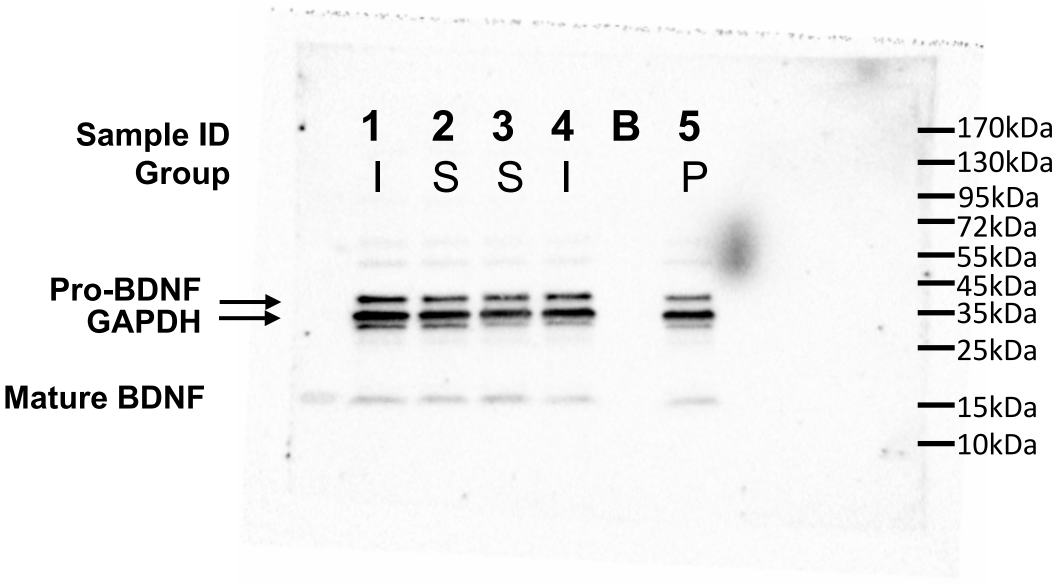

Supplement: S1 Raw images — Unedited raw images from each western blot used in densitometric analyses shown in Figs 4 and S7. (PDF) [file pone.0235566.s008.pdf]
