# Supplementary material for: Induction of fatigue-like behavior by pelvic irradiation of male mice alters cognitive behaviors and BDNF expression
Source: PLoS One. 2020 Jul 2;15(7):e0235566. doi: 10.1371/journal.pone.0235566 (PMC7332074; doi:10.1371/journal.pone.0235566)
Supplement: Attachment — Submitted filename: reviewer comments v01.docx [file pone.0235566.s010.docx]

**Reviewer #2**: While the authors have been responsive, the following changes are required to the figures:

1. Fig. 2. remove panels C, E, and F to suppl data as there is no significance; put the number of mice in the figure legend; in the G panel, show only the significant correlation and mention the other two in the text or figure legend.

2. Fig. 3. remove panels E, F, H, I, J to suppl data as there is no significance; put the number of mice in the figure legend; in the D panel, show only the two significant correlation and mention the third one in the text or figure legend.

3. Fig. 4. remove panel C to suppl data; panel D, show only the significant correlations; put the mouse numbers in the figure legend.

**Authors**: We have made the requested changes, moving non-significant results to supplementary material and removing non-significant correlation values (r and p values) from the figures (though they are still written in the main text of the Results section). We have added the number of mice to the figure legend of Fig 1, where we describe the experimental design; all other figures already show the number of mice (which was requested by one of the other reviewers in the previous round of comments). The number of mice is also written in the text of both the Methods section and in the Results section.

**Reviewer #4**: The authors have satisfactorily addressed all comments. There are two minor issues that require authors' attention:

1. Figure 3C, Y axis. This is normalized data (normalized to food pellets consumed on the day before irradiation). Consequently, the Y axis should be labeled as Food Pellets (% baseline).

**Authors**: Thank you for pointing this out. The Y-axis was correct, but the figure legend was wrong, and so we have corrected the legend. To be clear, the data are not normalized; mice often eat around 100 pellets per day.

2. Figure S5 D&E. Here the authors label Y axis as Open Time. Is this time in the center of the OF arena? If so, Center Time should be used to be consistent with labels used in Figures 2 and S2.

**Authors**: You are correct, and we have changed the axis label to “Center time” and expressed it properly as a percentage.

3. Figure S3. Can the authors provide a scale bar for the color scheme? The descriptive language "with darker colors representing more pellets, light colors representing fewer pellets" is not very helpful.

**Authors**: We agree, and we have added a scale bar to the all the figures that are in this format. We also reworded the figure legends to be more clear about what we measured.
